# A circadian rhythm-related biomarker for predicting prognosis and immunotherapy efficacy in lung adenocarcinoma

**DOI:** 10.18632/aging.204411

**Published:** 2022-12-01

**Authors:** Yuanjun Cheng, Jie Yao, Qianru Fang, Bin Chen, Guohui Zang

**Affiliations:** 1 Department of Cardiothoracic Surgery, People’s Hospital of Chizhou, Chizhou, China; 2 Department of Obstetrics, People’s Hospital of Chizhou, Chizhou, China

**Keywords:** circadian rhythm, immune checkpoint inhibitor, prognosis, lung adenocarcinoma, BARX2

## Abstract

Lung adenocarcinoma (LUAD) remains a major reason of cancer-associated mortality globally, and there exists a lack of indicators for survival in LUAD patients. Therefore, it is clinically required to obtain a novel prognostically indicator for guiding clinical management. In this study, we established a circadian rhythm (CR) related signature by a combinative investigation of multiple datasets. The newly-established signature showed an acceptable ability to predict survival and could serve as an independent indicator for prognosis. Moreover, the newly-established signature was critically associated with tumor malignancy, including proliferation, invasion, EMT and metastasis. The newly-established signature was predictive of response to immune checkpoint blockade. Collectively, we established a CR-related gene signature that could forecast survival, tumor malignancy and therapeutic response; our findings could help guiding clinical management.

## INTRODUCTION

Pulmonary carcinoma remains a major culprit for cancer-associated mortality globally. Lung adenocarcinoma (LUAD) represents a majority of all types of pulmonary carcinoma and has been elevating annually [1]. Though recent progress in the clinical management of LUAD, the overall prognosis of patients with LUAD remains comparatively low, less than 20% [2]. Currently, there is still a need for an improved prognostic indicator for survival in lung adenocarcinoma. Therefore, it is clinically required to obtain a novel indicator to forecast survival in LUAD.

Circadian rhythm (CR) is defined as a psychological cycle and regulate various physiobiological functions in creatures [3]. CR disturbance is reported to increase hazard of suffering from cancer, implying the impact of CR in human health. The roles of CR genes have been documented in the etiology of human diseases [3], and other types of cancer [4, 5]. At the same time, accumulating documents showed its relevance in the immune system [6–8]. Although CR has been one hotpot of cancer research field in recent years, the molecular mechanisms underlying its function are still incompletely understood. At present, circadian rhythm disruption is reported to promote lung tumorigenesis [3] and associated with poor prognosis and drug response [9]. Nevertheless, we still do not know if the CR-related biomarker could constitute an independent predictor for pulmonary cancer.

Identifying a novel indicator for pulmonary cancer, the present study analyzed gene expression profiling from the cancer genome atlas (TCGA) and gene expression omnibus (GEO) by computational algorithms. This study should afford us the opportunity to develop a new biomarker for forecasting prognosis for individual patients with pulmonary carcinoma.

## MATERIALS AND METHODS

### Public datasets

Gene expression profiles of 585 lung adenocarcinoma were downloaded from TCGA, including 526 lung adenocarcinoma samples and 59 normal lung samples. Among them, there are 524 primary tumor samples and 2 metastatic samples. Microarray expression data and corresponding survival data of 83 lung cancer samples was obtained from the GSE30219 cohort. There were 117 circadian rhythm-related genes, which come from the molecular signature database (MSigDB), used to select qualified candidate genes in the present study. Gene expression profiles were standardized into TPM and followed by log_2_(TPM + 1) transformation. Gene expression profiles from GEO were normalized using gcRMA algorithm.

### Identification of differentially expressed genes

We identified differentially expressed genes (DEGs) with edgeR in R [10]. The core algorithm of edgeR was based upon negative binomial distribution. The general pipeline was as follows: establishing a model to estimate the data distribution properties, performing empirical Bayes algorithm, and carrying out quasi-likelihood calculation. Screening criteria were |logFC| > 2 as well as FDR < 0.001 [11].

### Quantification of gene sets in individual samples

Single-sample gene set enrichment analysis (ssGSEA) [12] is a computational algorithm to quantify changes of a gene list in individual samples. The ssGSEA value means the relative expression level of a specific gene list in every individual.

### Establishment of the CR-associated signature

Among 526 LUAD patients from the TCGA cohort, there are 500 LUAD patients with a completed survival data. These 500 patients were stochastically categorized as the discovery cohort (n = 400) and validation cohort (n = 100).

The candidate gene list was fed into the least absolute shrinkage and selection operator (LASSO) regression. Then, genes with a *P* < 0.05 in LASSO were applied for establishing a signature. The hazard was quantified by the equation: TTC39C×(-0.133)+ BARX2×(0.042)+ BHLHA15×(-0.107)+ KCNQ3×(0.001)+ S100P×(0.038)+ DDIT4×(0.143)+ INHA×(0.064)+ CNGA3×(-0.001) + WFDC2×(-0.0971).

### Evaluation of the CR-associated biomarker

The prognostic gene signature was used to quantify the hazard of individual samples using the abovementioned method. The predictive performance of the prognostic gene signature was assessed using receiver operating characteristics curve and Kaplan-Meier curve in the discovery cohort, the validation cohort, and GSE30219.

### Functional annotation

Functional enrichment analyses of DEGs were conducted based on clusterProfiler in R language [13]. The clusterProfiler can investigate the biological function of a give gene list using the canonical gene sets in Gene Oncology (GO) as well as Kyoto Encyclopedia of Genes and Genomes (KEGG). Important parameters were set in default.

### Establishing of a nomogram for precisely predicting prognosis

Combining the forecasting value of the established signature with the commonly-utilized clinical features, we developed a nomogram, which could be used to precisely quantify the hazard of individual patients.

### Statistics

Statistics was achieved in RStudio (Version 4.0.1). Independent sample *t* test or Wilcoxon signed rank test was applied to compare two continuous variables. The capacity of the prognostic signature was evaluated by receiver operating characteristic curve (ROC) and Kaplan-Meier curve. Spearman’s rank correlation coefficient was applied to evaluate the association between two continuous variables. *P* < 0.05 was recognized statistically relevant.

### Consent to publish

The authors agree to be published in this magazine.

### Availability of data and materials

All data generated and described in this article are available from the corresponding web servers, and are freely available to any scientist wishing to use them for noncommercial purposes, without breaching participant confidentiality. Further information is available from the corresponding author on reasonable request.

## RESULTS

### Construction of a novel gene signature

**Figure 1:**
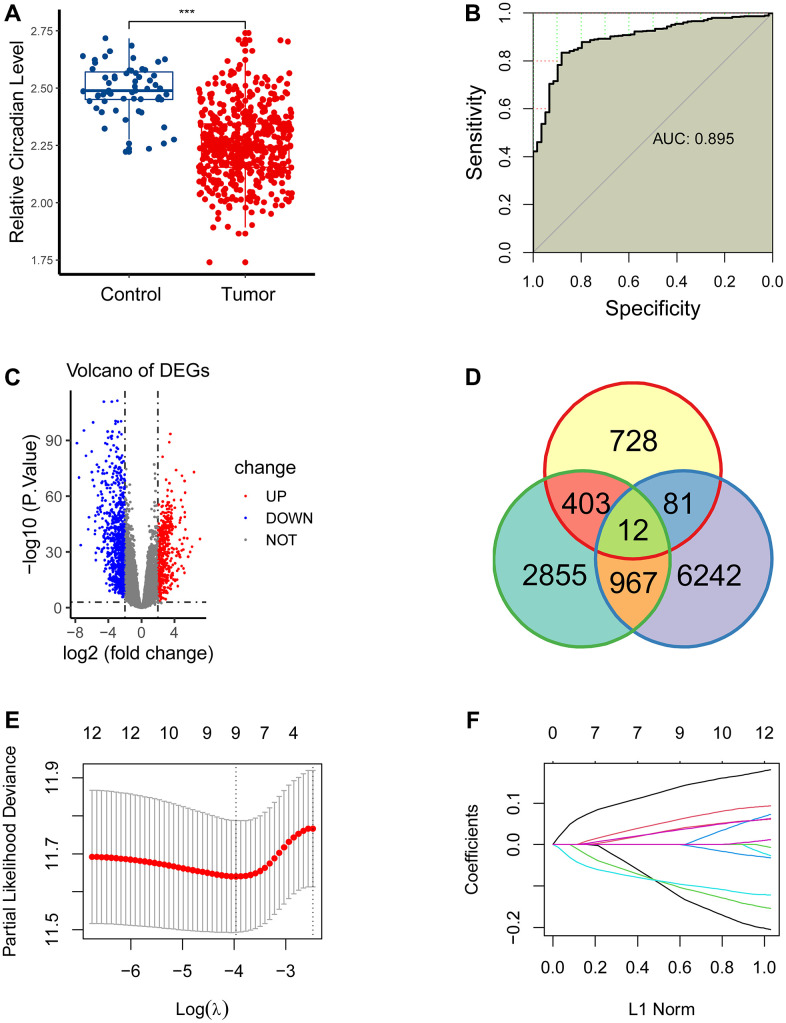
**Construction of a novel gene signature.** (**A**) CR levels were significantly upregulated in the para-cancerous samples compared to the tumor samples. (**B**) CR levels could distinguish tumor patients with normal patients, with area under curve (AUC) of 0.895. (**C**) 1224 differentially expressed genes (DEGs) were obtained between the paracancerous and tumor groups. (**D**) 12 eligible CR-related genes were acquired by intersecting 1224 DEGs, 4237 prognostically relevant genes and 7302 CR-related genes. (**E**, **F**) 12 eligible CR-genes were further screened using LASSO regression analysis.

CR has been linked to human diseases, however its function on LUAD is to be interrogated. Here, we interrogated the CR levels between the LUAD and para-cancerous samples using RNA-seq data of LUAD patients. The CR levels were estimated using the ssGSEA approach, and we found that CR levels were significantly upregulated in the para-cancerous samples compared to the tumor samples (Figure 1A). Moreover, we performed receiver operating characteristics curve for circadian rhythm signaling pathway, and observed that the CR levels could distinguish tumor patients with normal patients, with area under curve (AUC) of 0.895 (Figure 1B).

We next planned to interrogate the role of CR in the prognosis of LUAD. First, we conducted differential expression analysis to get 1224 DEGs between the para-cancerous and tumor cohorts (Figure 1C), conducted a log-rank test for all genes to acquire 4237 prognostically relevant genes (P<0.05), carried out correlation analysis to obtained 7302 CR-related genes (*P* < 0.05, *R* > 0), and eventually get 12 eligible CR-related genes by intersecting 1224 DEGs, 4237 prognostically relevant genes and 7302 CR-related genes (Figure 1D).

We then screened 12 CR-genes with LASSO to remove multicollinearity. Eventually, nine CR-related genes (*TTC39C*, *BARX2*, *BHLHA15*, *KCNQ3*, *S100P*, *DDIT4*, *INHA*, *CNGA3*, *WFDC2*) were selected for the establishment of a CR-related biomarker (Figure 1E, 1F).

### Assessment of predictive capability of the newly-established biomarker

**Figure 2:**
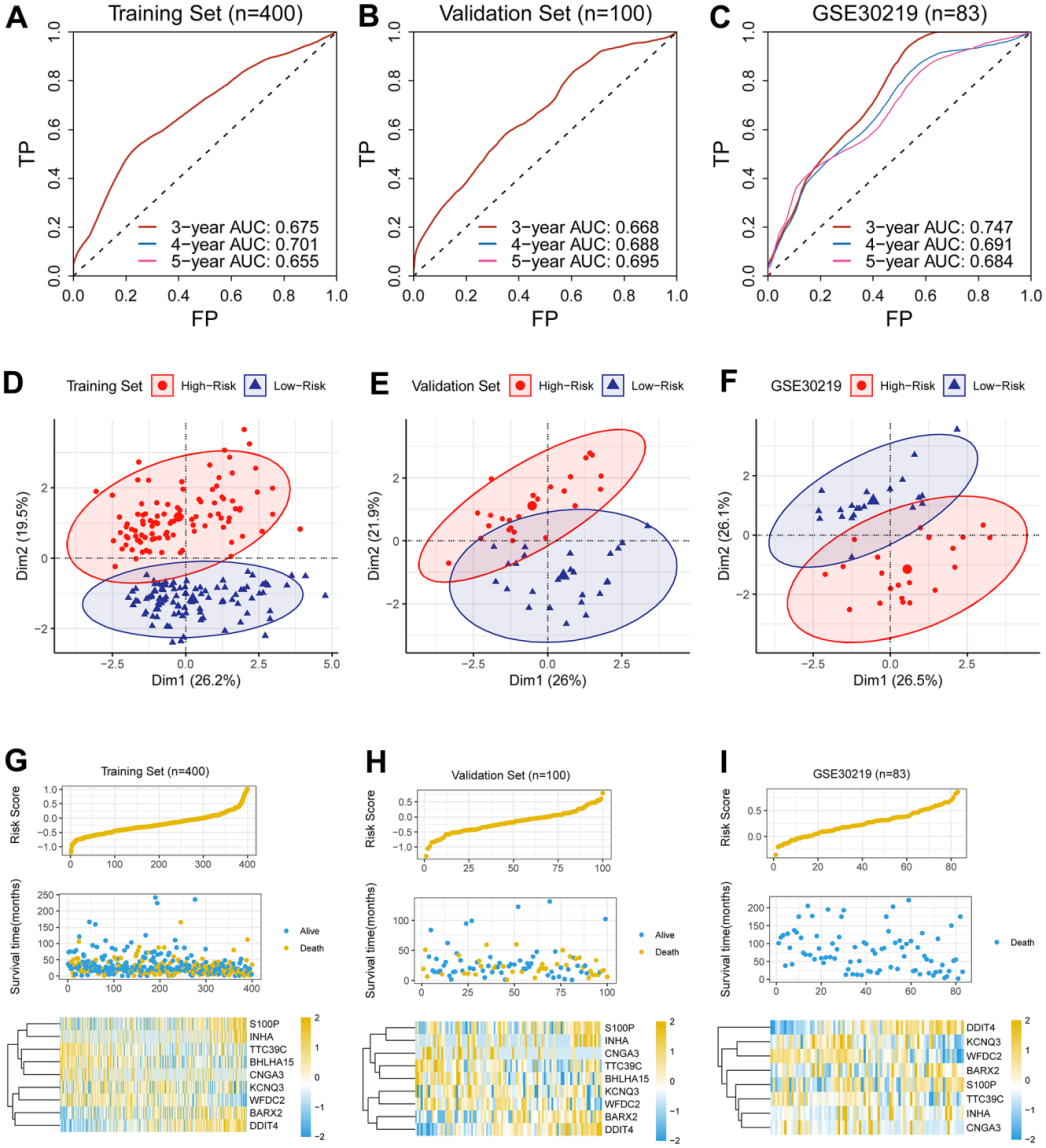
**Assessment of predictive capability of the newly-established biomarker.** (**A**–**C**) Three-year AUCs were 0.701, 0.688 and 0.691 in the discovery cohort, the validation cohort and GSE30219, respectively. (**D**–**F**) PCA analysis revealed that the low-risk group was apparently separated from its counterpart in dimensionality 1. (**G**–**I**) The hazard was linked to unwanted prognosis and high expression levels of four genes (*BARX2, DDIT4, INHA, S100P*) in the discovery cohort, the validation cohort and GSE30219.

The capability of the CR-related signature was assessed in the discovery cohort (n = 400), the validation cohort (n = 100) and GSE30219 (n = 83). Three-year AUCs were 0.701, 0.688 and 0.691 in the discovery cohort, the validation cohort and GSE30219, respectively (Figure 2A–2C), suggesting its robust predictive ability for predicting prognosis.

We quantified the hazard for individual patients and divided the patients into the high- and low-risk cohorts. PCA analysis revealed that the low-risk cohort was apparently separated from its counterpart in dimensionality 1 (Figure 2D–2F), indicating the CR-related gene signature has a good distinguishing ability.

In addition, we analyzed the relationship between the survival status, the survival risk, and the mRNA levels; we observed that the hazard was linked to unwanted prognosis and high expression levels of four genes (*BARX2, DDIT4, INHA, S100P*) in the discovery cohort (Figure 2G), the validation cohort (Figure 2H) and GSE30219 (Figure 2I).

### Clinical significance of the novel gene signature

**Figure 3:**
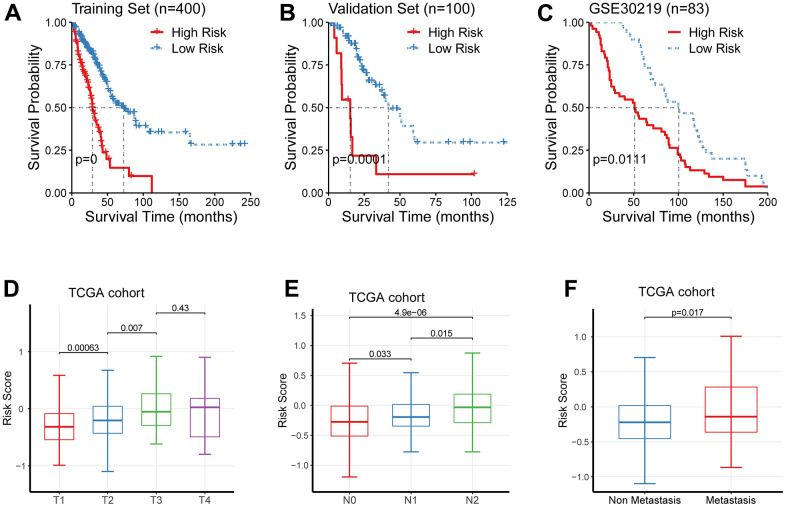
**Clinical significance of the newly-established signature.** (**A**–**C**) The low-risk cohort showed an improved prognosis than its counterpart in the discovery cohort, the validation cohort, and GSE30219. (**D**–**F**) The risk levels were significantly augmented in patients of higher TNM staging.

Since the signature showed an acceptable forecasting capability, we next sought to investigate its clinical relevance. As previously described, we quantified the hazard of individual patients, and categorized them as the low- and high-risk cohorts. In line with the results generated from the previous step, the low-risk cohort showed an improved outcome than its counterpart in the discovery cohort, the validation cohort, and GSE30219 (Figure 3A–3C).

We further interrogated the link between the newly-established signature and TNM staging, and observed that the risk levels were significantly augmented in patients of higher TNM staging (Figure 3D–3F).

### Development of a prognostic nomogram

**Figure 4:**
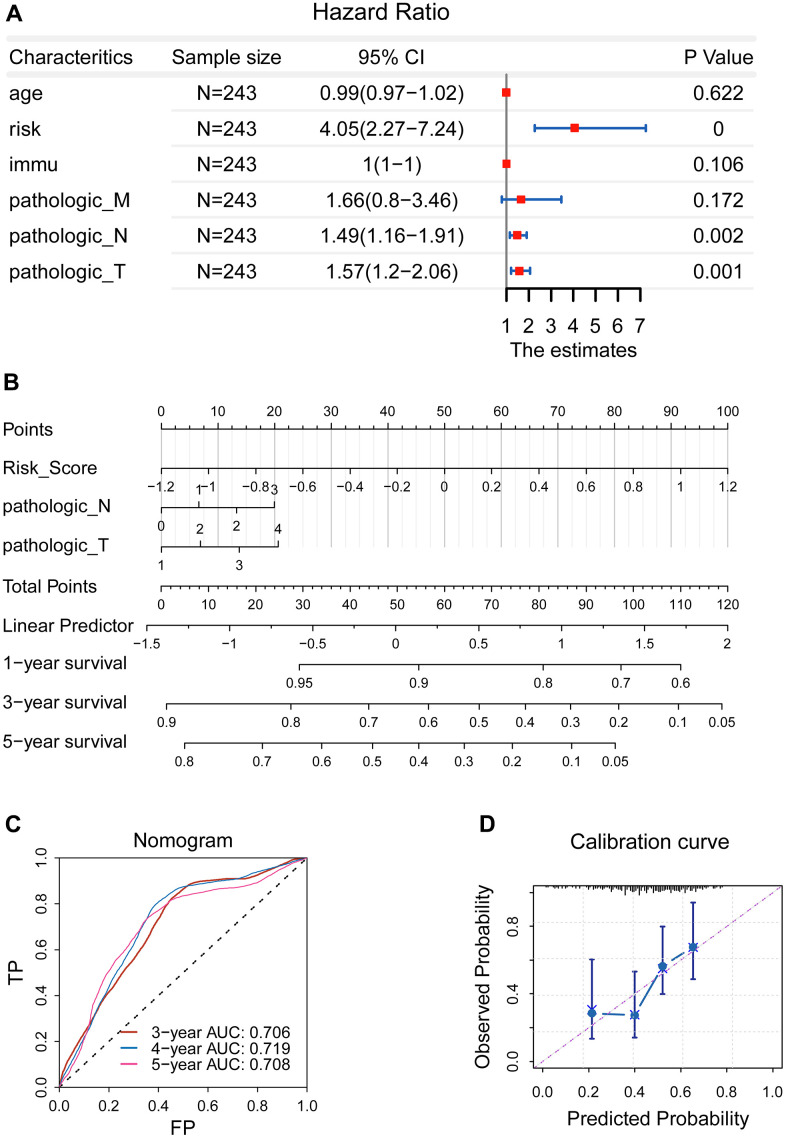
**Development of a prognostic nomogram.** (**A**) Pathological N, the pathological T, and the newly-established signature were selected as effective indicators. (**B**) A nomogram was constructed. (**C**) The ROC curve demonstrated an elevated forecasting performance, and five-year AUC value was 0.708 (**D**) Calibration curve revealed that the calculated probability line was highly compatible with observed probability line.

Next study continued to examine the prognostic significance of the newly-established signature by means of univariate Cox regression. Pathological N, the pathological T, and the newly-established signature were selected as effective indicators (Figure 4A). We noticed that pathological M showed no significant prognostic value (*P* = 0.172), which seems to be contrary to the current knowledge that patients with pathological M have a poor survival. Thus, we further analyzed the clinical data and found that there were 229 patients with M0 while 14 patients with M1. In other words, the sample size between the M0 and M1 cohorts was extremely imbalanced and thus affect the statistical results.

Afterwards, we constructed a prognostic nomogram combining the risk score, pathological N, and pathological T (Figure 4B), which can be used to accurately predict the patients’ survival risk for precision medicine by calculating specific survival risk scores based on the risk score, pathological N, and pathological T. The ROC curve demonstrated an elevated forecasting performance, and five-year AUC value was 0.708 (Figure 4C). Accordingly, calibration curve revealed that the calculated probability line was highly compatible with observed probability line (Figure 4D).

### Functional analysis

**Figure 5:**
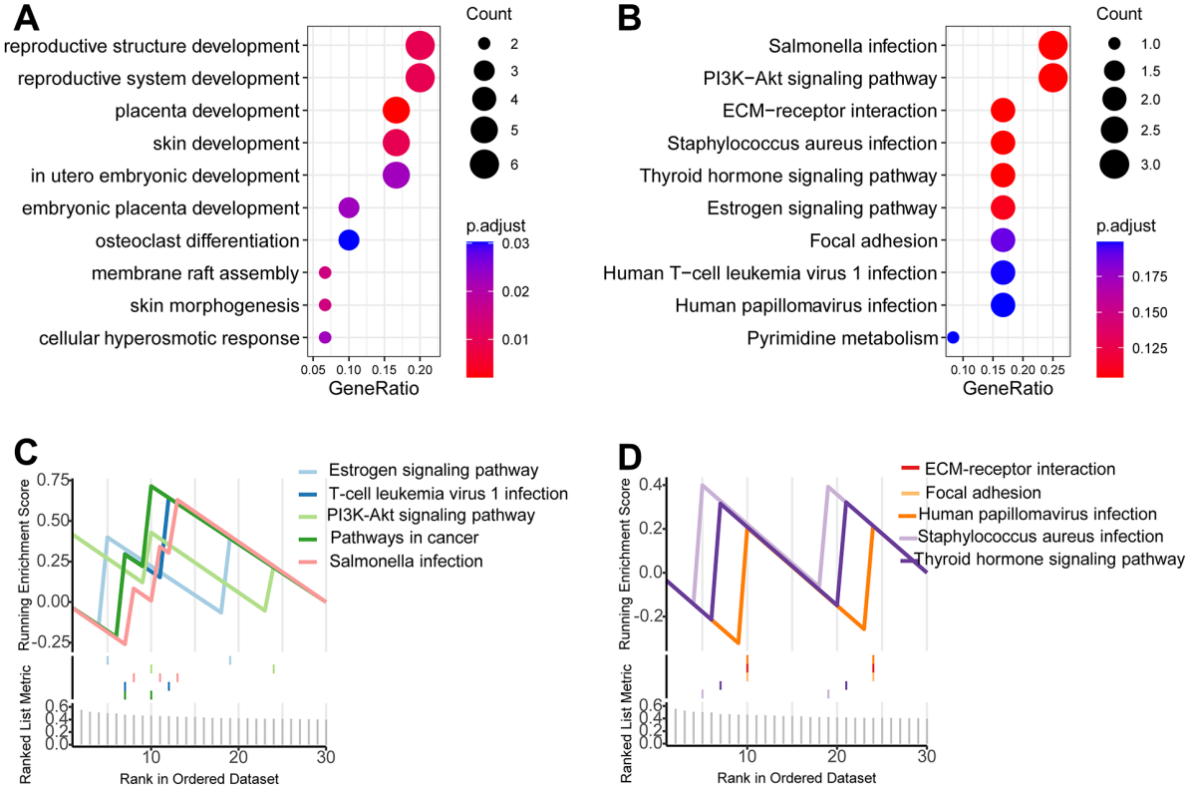
**Functional analysis.** (**A**, **B**) Gene oncology analysis showed that multiple cancer-associated pathways were identified. (**C**, **D**) GSEA showed multiple cancer-associated pathways were significantly enriched in the high-risk patients.

We first investigated the relationship of the newly-established signature with molecules using gene expression profiling. 123 genes were acquired as candidate genes. The 123 candidates were analyzed using clusterProfiler in R language. The findings showed that multiple cancer-associated pathways were identified (Figure 5A, 5B). Consistently, GSEA showed multiple cancer-associated pathways were significantly enriched in the high-risk patients (Figure 5C, 5D).

### Relationship between the newly-established signature with cancer behaviors

**Figure 6:**
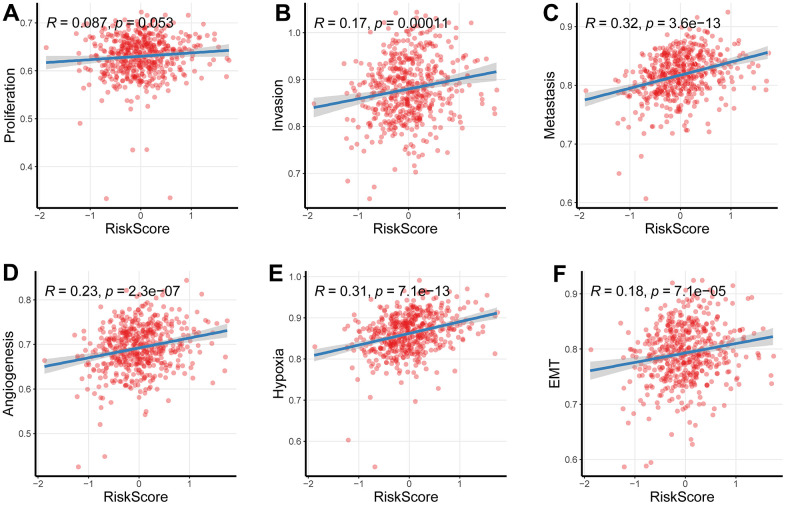
**Relationship between the newly-established signature with cancer behaviors.** (**A**–**C**) The signature was linked to these malignant tumor behaviors including invasion and metastasis, while its relationship with proliferation was comparatively weak. (**D**–**F**) The newly-established signature was implicated into the levels of hypoxia, EMT as well as angiogenesis (*P* < 0.05).

Considering predictive capacity of the newly-developed gene signature, we wondered whether this signature could reflect the malignant degree. For this purpose, we estimated enrichment score for tumor malignant behaviors using the ssGSEA algorithm. Then we analyzed the relationship of the enrichment score of proliferation, invasion and metastasis with the risk scores based on CR-related gene signature; the findings indicated that the newly-established signature was linked to these malignant tumor behaviors including invasion and metastasis (Figure 6B, 6C), while its relationship with proliferation was comparatively weak (Figure 6A).

Investigating the underlying mechanisms of the effects of the newly-established signature on cancer behaviors, we further assessed the association between the newly-established signature and hypoxia, epithelial-mesenchymal transition (EMT) and angiogenesis, which are all the potential causes of tumor metastasis. As we expected, the newly-established signature was implicated into the levels of hypoxia, EMT as well as angiogenesis (Figure 6D–6F).

### Identification of candidate CR-related hub genes in LUAD

**Figure 7:**
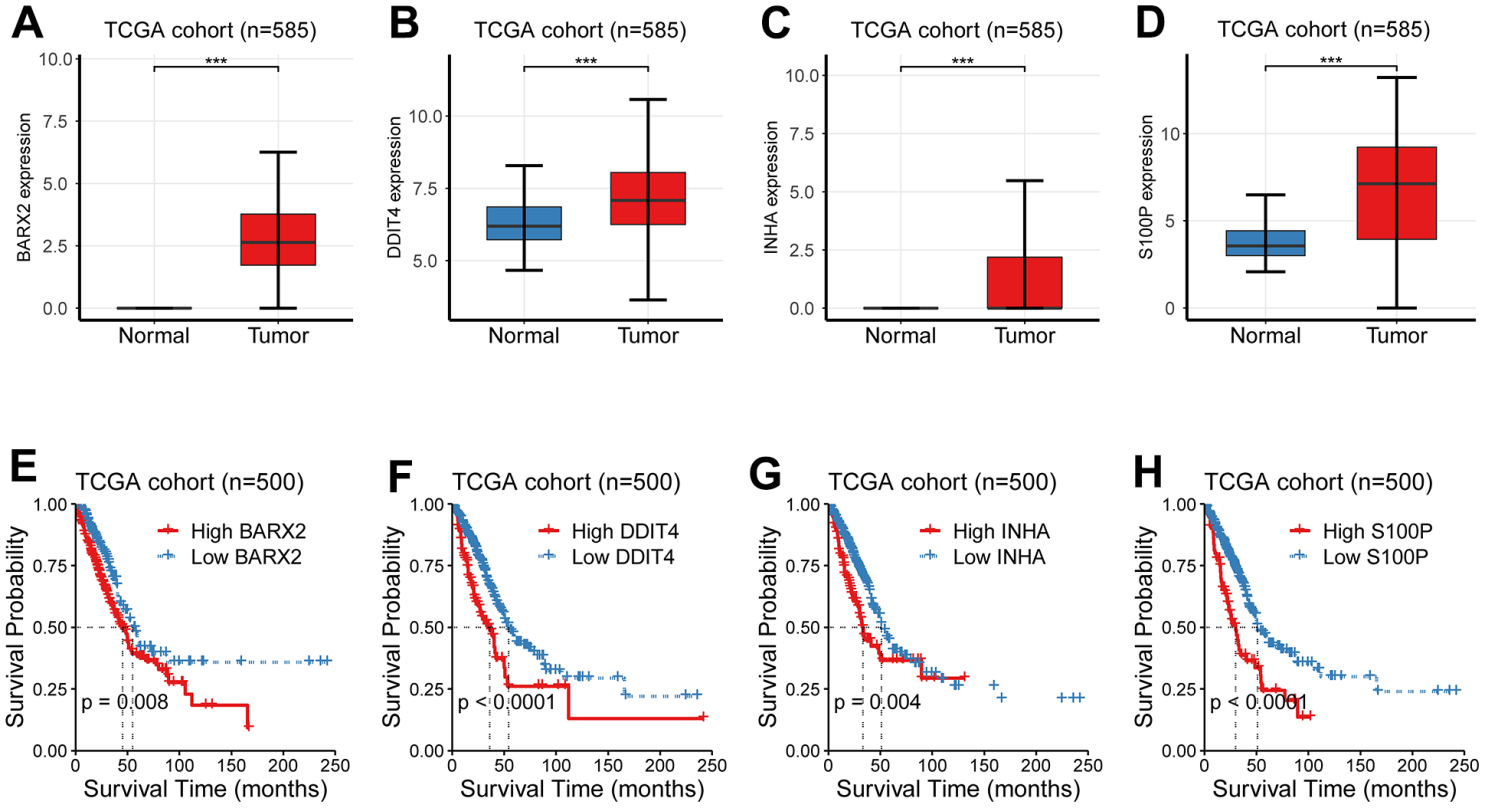
**Identification of candidate CR-related hub genes in LUAD.** (**A**–**D**) The expression levels between the para-cancerous and cancer samples showed an apparent distinction. (**E**–**H**) Four CR-related genes (*BARX2, DDIT4, INHA, S100P*) had a survival significance.

Considering the above established nine-gene signature not only can reflect overall survival, but also was associated with TNM staging, we speculated that these nine genes were candidate critical genes in the development of LUAD. We first compared their expression levels between the para-cancerous and cancer samples and observed an apparent distinction between the para-cancerous and cancer groups (Figure 7A–7D and Supplementary Figure 1). Then, we investigated their survival significance and revealed that these nine molecules were all had a survival significance (Figure 7E–7H and Supplementary Figure 1). Noticeably, four upregulated genes (*BARX2, DDIT4, INHA, S100P*) in LUAD showed a significantly reduced survival, suggesting their potential role in the development of LUAD.

### The investigation of somatic mutation between the low-risk cohort and high-risk cohort

**Figure 8:**
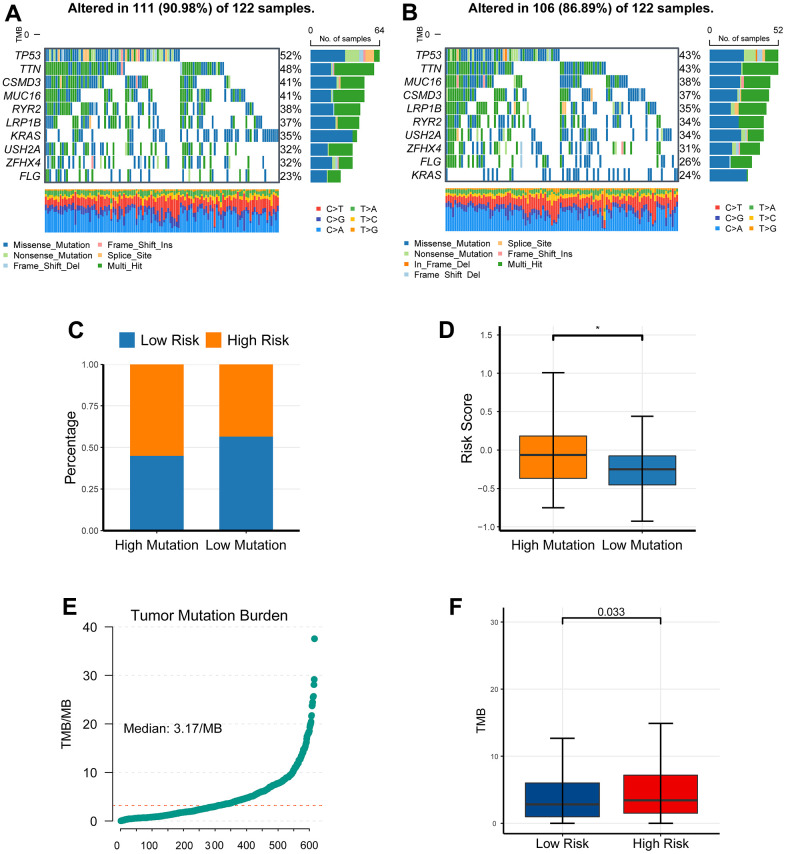
**The investigation of somatic mutation between the low-risk cohort and high-risk cohort.** (**A**, **B**) The waterfall plot showed a distinct mutation rate between the low- and the high-risk cohorts. (**C**, **D**) The high mutated cohort had a higher risk than the low mutated cohort. (**E**) TMB was estimated with maftool in R, and ranged from 0.02/MB to 37.54/MB. (**F**) TMB was significantly elevated in the high risk cohort compared to its counterpart. *P <0.05, **P <0.01, ***P <0.001.

Comparing the difference of the mutation profiles between distinct risk cohorts, we set the top quarter patients as the high risk cohort and set the bottom quarter patients as its counterpart. The waterfall plot showed a distinct mutation rate between the low- and the high-risk cohorts (Figure 8A, 8B). Consistently, further analysis demonstrated that the high mutated cohort had a higher hazard than the low mutated cohort (Figure 8C, 8D). Tumor mutation burden (TMB) is recognized to be linked to immune checkpoint blockade [14, 15]; therefore, we analyzed the association between the hazard and TMB. TMB was estimated with maftool in R, and ranged from 0.02/MB to 37.54/MB (Figure 8E). In accordance with our expectation, TMB was significantly elevated in the high hazard cohort compared to its counterpart (Figure 8F).

### The impact of the newly-established signature on immunotherapy

**Figure 9:**
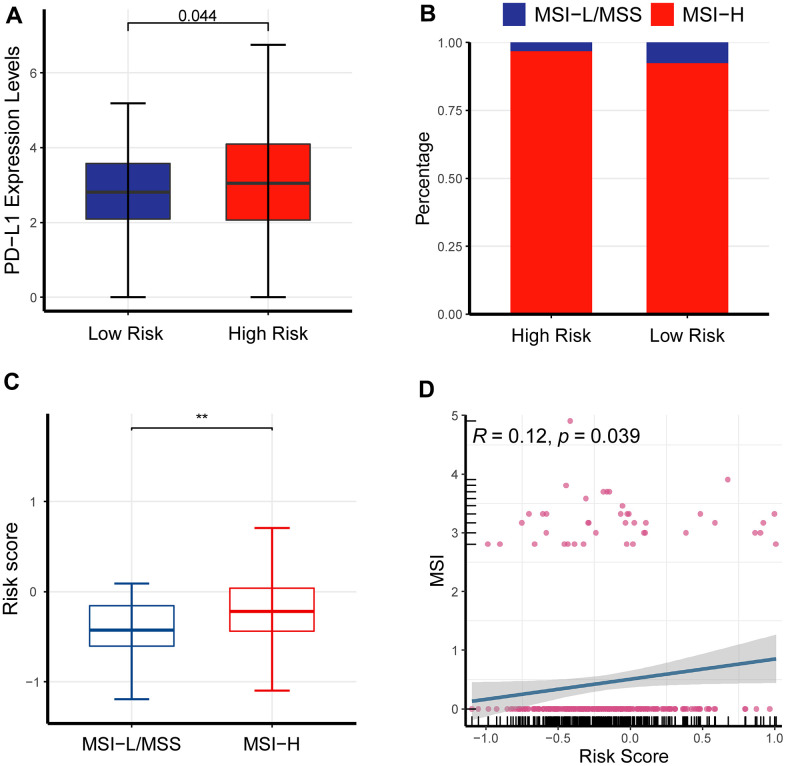
**The impact of the newly-established signature on immunotherapy.** (**A**) PD-L1 was overexpressed in the high-risk cohort than its counterpart. (**B**) Bar plot showed MSI-H had a larger fraction in the high-risk cohort than its counterpart. (**C**) Box plot showed that the high-risk cohort had an elevated MSI compared to its counterpart. (**D**) Scatter plot demonstrated that MSI was critically associated with risk scores.

TMB, high-microsatellite instability (MSI-H) as well as PD-L1 have been associated with response to immune checkpoint blockade [16]. Herein, PD-L1 was overexpressed in the high-risk cohort than its counterpart (Figure 9A), implying the forecasting capability of the newly-established signature.

MSI levels of LUAD patients were quantified with two different methods, including UCSCXenaShiny and PreMSIm in R language, respectively. Bar plot showed MSI-H had a larger fraction in the high-risk cohort than its counterpart (Figure 9B). Box plot and scatter plot showed a consistent result (Figure 9C, 9D).

## DISCUSSION

The present study developed and validated a nine-gene indicator for forecasting survival in LUAD patients and identified multiple cancer-related signaling pathways. In addition, this study demonstrated that the newly-established predictor was linked to TMB, PD-L1 and MSI, as well as immunotherapy in LUAD. Conclusively, these findings supplied an acceptable biomarker to forecast prognosis of LUAD patients.

One main contribution represents the establishment of a newly-established predictor for LUAD patients. Robustness of the predictor was verified in a publicly available dataset, which makes the gene signature highly reliable and widely applicable. The predictive power of this gene signature is superior to other known gene signatures. Although the performance of the nine-gene signature was not statistically compared with other similar gene signatures for LUAD, we found that the performance of this CR-related gene signature is better than other similar markers [17–20]. Meanwhile, another strength of our signature is that our signature has been validated in an independent external dataset, which could ensure the reliability and broad applicability of our signature.

We also revealed that four CR-related oncogenes (*BARX2, DDIT4, INHA, S100P*), which could be used as potential therapeutic targets. We revealed that these four genes were overexpressed in cancerous tissue compared with norm lung tissue and linked to an unwanted outcome in LUAD cohort. The protein encoded by *BARX2* is implicated in actin rearrangement in lymphocyte spreading and immune synapse formation [14, 15]. Moreover, *DDIT4* was identified to be an ideal molecule to classify patients with skin melanoma [16], and related to poor overall survival in lung adenocarcinoma patients [21]. Blockade of pleckstrin-2 restores its negative effects [22]. *INHA* is also involved in cancer invasion and metastasis in various types of cancer [23–25]. Consistent with our findings, *S100P* is also identified to be a predictor of stomach tumor [26], and is essential for tumor aggressiveness in various types of cancer [27–29]. Here, we revealed its overexpression and correlation with poor survival outcomes in lung adenocarcinoma, pointing to the importance of further investigating its effects on the pathogenesis of pulmonary carcinoma.

Another important finding here is that several important biological processes, which are involved in CR and poor prognosis, were identified. Such biological processes have been found to cause regrowth and healing of tissue and aggressive cancerous behaviors [30]. Cancer-associated fibroblasts account for a large proportion of the ECM components within tumor [31, 32]. ECM diverges significantly between tumor and normal tissue. Intra-tumoral signaling pathways, metabolisms, oxygenation, and immunogenicity are critically influenced due to ECM. Therefore, ECM affects both the tumor expansion and aggressiveness and therapeutic effects [30]. Targeting ECM could be an alternative cancer therapy strategy [33, 34].

The present research owns some applicable significance. We developed a novel predictor for LUAD. Then, this study provided some protumor molecules in LUAD. On the other hand, the present research holds some shortcomings. The pivotal molecules are required to be validated *in vitro* and *in vivo*. Cell cycle, DNA replication, chromosome segregation, and nuclear division are indicated to therapeutic targets for the treatment of LUAD, while their mechanisms is to be investigated.

To summarize, a novel CR-related signature was established and evaluated, which could precisely forecast survival. We also revealed the important function of several critical genes, which may exert a role in designing new therapeutic strategies in LUAD.

## Supplementary Material

Supplementary Figure 1
