# Supplementary material for: A circadian rhythm-related biomarker for predicting prognosis and immunotherapy efficacy in lung adenocarcinoma
Source: Aging (Albany NY). 2022 Dec 1;14(23):9617–31. doi: 10.18632/aging.204411 (PMC9792196; doi:10.18632/aging.204411)
Supplement: Supplementary Figure 1 [file aging-14-204411-s001.pdf]

SUPPLEMENTARY FIGURE

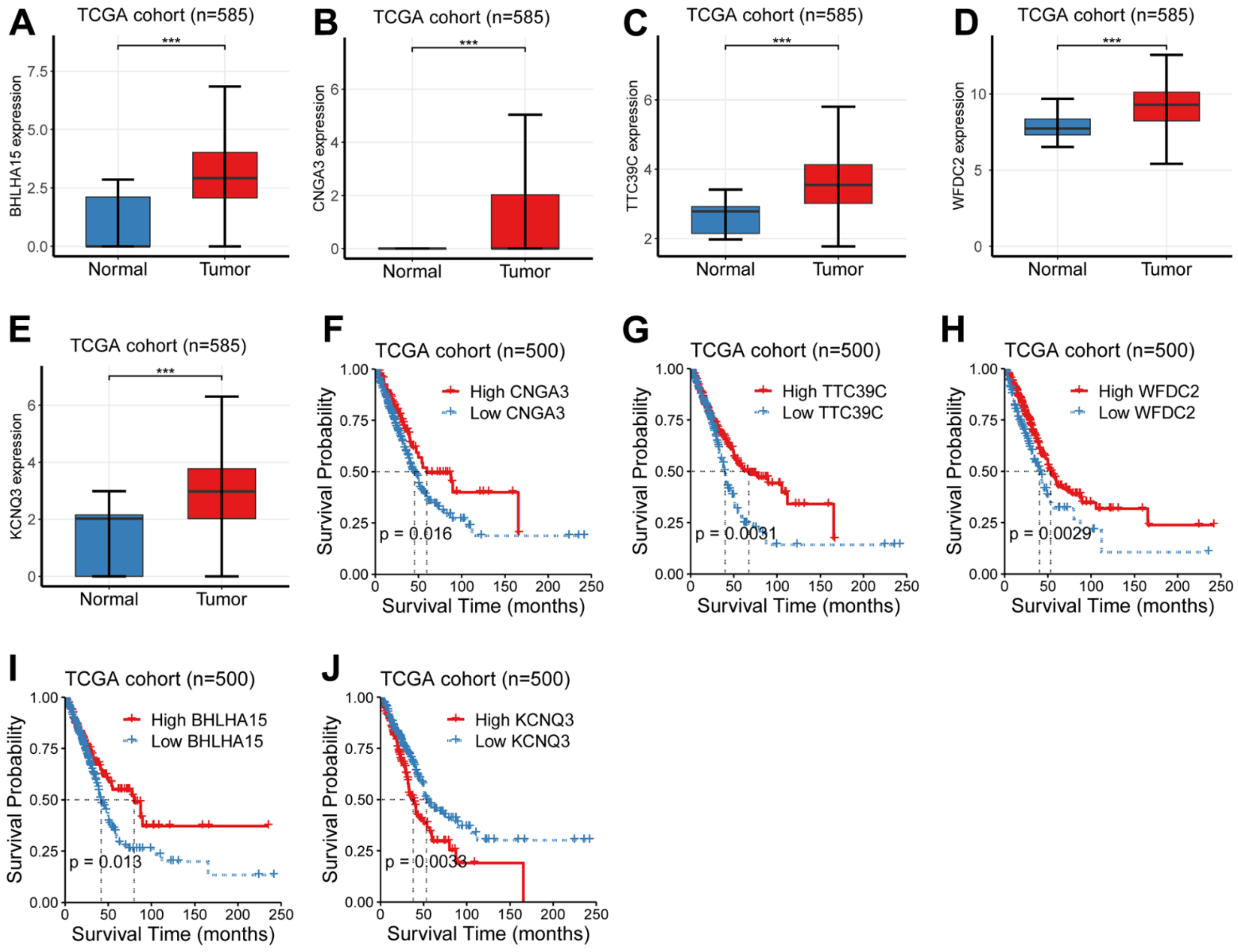

Supplementary Figure 1. Investigation of the candidate CR-related genes in LUAD. (**A**–**E**) Box plots that display the gene expression levels of the CR-related genes. (**F**–**J**) Kaplan-Meier curve that demonstrate the survival difference between the low- and high-group.
